# Maine Veterinary Medical Association

**Published:** 1900-03

**Authors:** I. L. Salley

**Affiliations:** Secretary


Secretary.

### MAINE VETERINARY MEDICAL ASSOCIATION.

THE regular annual meeting was held at Hotel North, Augusta, Wednesday, January 10th, at 7.30 P.M. A small number of members were present. The minutes of the previous meeting were read and accepted.

Dr. A. Joly, for the Committee on Clinics, reported that all the cases operated on at the meeting in October were successful except one case of spavin, which was cauterized.

Election of officers resulted as follows: Dr. A. Joly, Waterville, President; Dr. C. L. Blakely, Augusta, Vice-President. Dr. I. L. Salley was re-elected Secretary, but declined to serve, and Dr. F. E. Freeman, of Rockland, was elected. Dr. I. L. Salley was elected Treasurer. Drs. Russel, Murch, and Cleaves were appointed as the Executive Committee.

Dr. Blakely reported a case of angioleucitis. Dr. Salley reported a case of purpura hemorrhagica followed by trismus to an extent that the patient was unable to eat much but slops, and is in this condition at the present time. Considerable discussion followed the report of these cases.

Drs. Russel and Murch were appointed to read papers at the next meeting. Voted to adjourn to meet at Bangor in April.

I. L. SALLEY,
Secretary.

